# Association between healthy beverage index and nonalcoholic fatty liver disease in the Ravansar noncommunicable disease cohort study

**DOI:** 10.1038/s41598-024-54288-2

**Published:** 2024-02-13

**Authors:** Sepehr Sadafi, Ali Azizi, Shahab Rezaeian, Yahya Pasdar

**Affiliations:** 1 Clinical Research Development Center, Imam Reza Hospital, Kermanshah University of Medical Sciences, Kermanshah, Iran; 2 Social Development and Health Promotion Research Center, Kermanshah University of Medical Sciences, Kermanshah, Iran; 3 Department of Community and Family Medicine, School of Medicine, Kermanshah University of Medical Sciences, Kermanshah, Iran; 4 Research Center for Environmental Determinants of Health (RCEDH), Health Institute, Kermanshah University of Medical Sciences, Kermanshah, Iran

**Keywords:** Healthy beverage index, Fatty liver, Beverage quality, Diseases, Endocrinology, Risk factors

## Abstract

The quality of drinks affects the functioning of the liver. In recent decades, the variety of high-calorie and sweet drinks has increased. The objective of this study was to explore the association between Healthy Beverage Index (HBI) and the risk of nonalcoholic fatty liver disease (NAFLD) among adults. We included 6,276 participants aged 35 to 65 from the Ravansar Non-Communicable Disease (RaNCD) cohort study at baseline. NAFLD is defined based on the fatty liver index (FLI), calculated using anthropometric measurements and non-invasive markers. The HBI was developed using a combination of water, low-fat milk, 100% fruit juice, sugar-sweetened beverages, met fluid requirement and % energy from beverages. Logistic and linear regression models were employed to investigate the associations of the HBI and high FLI. The average FLI was significantly lower in the first tertile of HBI compared to the third tertile (47.83 vs. 45.77; *P* = 0.001). After adjusting for confounding variables, the odds of high FLI decreased by 28% (OR 0.72, 95% CI 0.63, 0.82) in the second tertile of HBI and by 21% in the third tertile (OR 0.79, 95% CI 0.70, 0.91). There was no correlation between gamma glutamyl transferase (GGT), alanine aminotransferase (ALT), alkaline phosphatase (ALP) and aspartate transaminase (AST) levels with HBI. The study findings indicate an inverse association between high FLI and HBI. Therefore, it is recommended to consume healthy beverages and without added sugar. However, additional longitudinal studies are required to examine the association between beverage consumption and the development of NAFLD.

## Introduction

Nonalcoholic fatty liver disease (NAFLD) is a rapidly growing global health concern. Various lifestyle factors, such as sedentary behavior, high fat and sweet diet, easy access to processed foods and obesity, have been identified as significant risk factors for the development of NAFLD^1–4^. The global incidence of NAFLD is estimated to be 47 cases per 1000 population, with a higher prevalence among men compared to women. The overall prevalence of NAFLD among adults worldwide is 32% (40% in men and 26% in women)^5^. In Asia, the prevalence of NAFLD varies due to the diverse ethnicities and socioeconomic factors across different countries. In Iran, the prevalence of NAFLD has been reported to be 38.07%^6^.

Diet plays a crucial role in NAFLD, as supported by numerous studies^2^. Especially, the quantity and quality of beverages have been recognized as important factors in daily dietary intake, influencing cardio-metabolic factors^7–9^. Research has consistently shown that the consumption of sugar-sweetened beverages (SSBs) is associated with weight gain and obesity^10^. Conversely, reducing SSBs intake has been linked to improvements in blood pressure, body weight, as well as a decreased risk of diabetes and cardiovascular diseases (CVDs)^11,12^. A study conducted in the US revealed a potential association between higher consumption of SSBs and the risk of liver cancer^13^. Analysis of data from the Framingham study further demonstrated that increased mean SSBs consumption over a 6-year follow-up period was associated with elevated liver fat levels and a higher incidence of NAFLD, particularly among older age groups^14^. The findings of the study conducted by Chhimwal J et al. indicate that drinking sugar-rich beverages raises the risk of developing NAFLD, whereas consuming coffee and tea notably decreases this risk^15^.

The Healthy Beverage Index (HBI) serves as a valuable tool for nutritionists and therapists to evaluate beverage quality and encourage the adoption of healthier beverage choices^9^. This index encompasses a range of common beverages such as water, milk, SSBs, tea & coffee, natural fruit juice, and the percentage of energy derived from beverages. It is indeed important to investigate the association between NAFLD and HBI specifically among Iranian adults, as no previous studies in Iran have explored this association. Given the variation in dietary patterns across different regions, it was deemed necessary to conduct this study on a large population of Iranians. Therefore, the objective of the current study was to examine the potential association between HBI scores and the high FLI risk among adults in western Iran.

## Methods

### Participants

**Figure 1:**
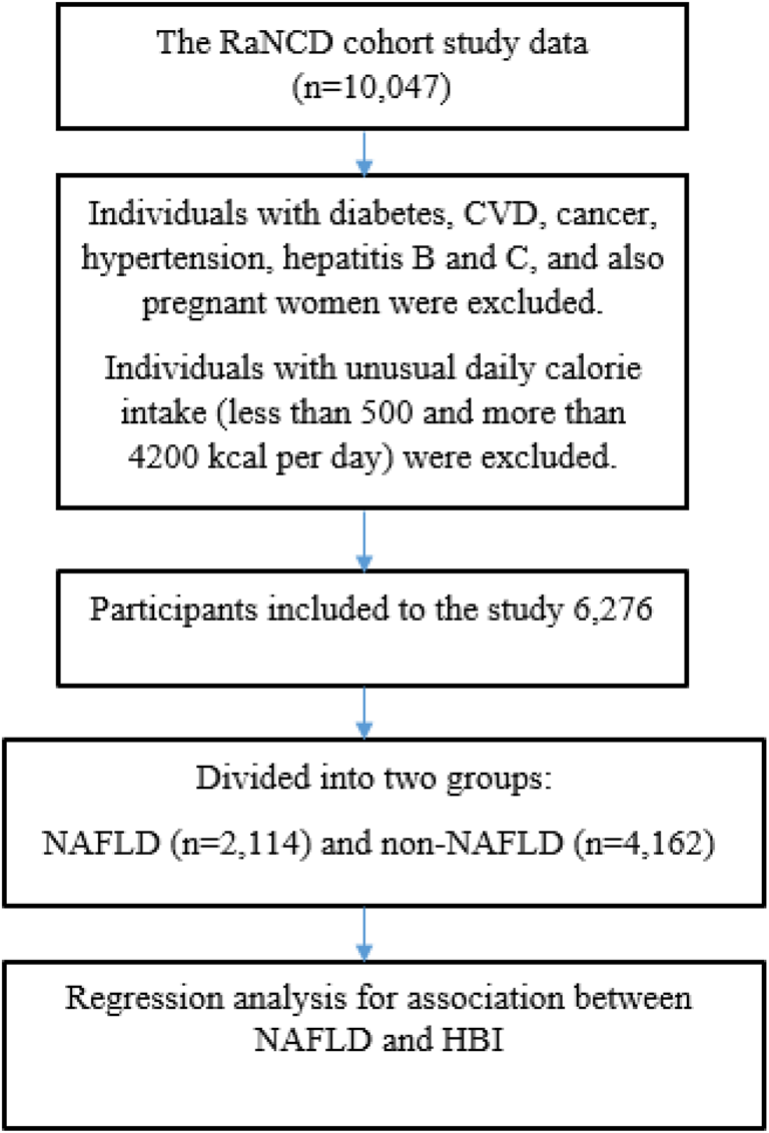
Flowchart of the study participants and data preparation.

This research involves analyzing data from the initial phase of the Ravansar Non-Communicable Disease (RaNCD) cohort study, which is a part of the PERSIAN (Prospective Epidemiological Research Studies in Iran) researches^16^. The baseline phase of the RaNCD study commenced in 2014 and included 10,000 adults aged 35–65, residing in urban and rural areas of Ravansar in western Iran. The participants were of Kurdish ethnicity. The study protocol for the RaNCD cohort has been previously published with additional details^17^. All participants from the baseline phase of the RaNCD study were included in this analysis, and after applying exclusion criteria, 6276 participants were assessed as depicted in Fig. 1**.**

### Data collection and measurements

All data were gathered in accordance with the cohort study protocol. Trained professionals utilized digital questionnaires of the cohort system to collect questionnaire information. Demographic details, including age, gender, and place of residence, were obtained. Socio-economic status (SES) was determined based on 28 variables such as education level, place of residence, prosperity, and wealth using the principal component analysis (PCA) method. The questionnaire also encompassed information on behavioral habits such as smoking and physical activity. Current smokers were defined as individuals who smoke at least 100 cigarettes per year. Physical activity was assessed using 22 questions about sports, work, and leisure activities over a 24-h period by the unit of MET/hour per day^17^.

The lipid profile, comprising triglycerides (TG), total cholesterol (TC), high-density lipoprotein cholesterol (HDL-C), low-density lipoprotein cholesterol (LDL-C), and fasting blood sugar (FBS), along with liver enzymes including Gamma-glutamyl transferase (GGT), alanine aminotransferase (ALT), alkaline phosphatase (ALP), and aspartate transaminase (AST), was assessed by collecting 25 cc of blood from the participants. As per the protocol, participants were instructed to fast for 8–12 h prior to the blood collection.

The participants' height was measured with a precision of 0.1 cm using a BSM 370 automatic stadiometer (Biospace Co., Seoul, Korea). To measure height, the person stood next to the wall without shoes, with their heels together and touching the wall, knees straight, and shoulders in a normal position. Body composition, including body mass index (BMI), visceral fat area (VFA), waist circumference (WC), and body fat mass (BFM), was assessed using a Bio-Impedance Analyzer BIA (Inbody 770, Inbody Co, Seoul, Korea). The participants stood on the device without socks and wearing minimal clothing. Additionally, they were asked not to carry any metal items, such as watches and keys.

FLI, introduced by Bedogni et al. in 2006, is an index used to evaluate the liver status^18^ according to the following formula:

$${\text{FLI}}=\frac{({{\text{e}}}^{ 0.953\times {\text{log}}({\text{e}}) ({\text{TG}}) + 0.139\times \mathrm{ BMI}+ 0.718\times \mathrm{ log }({\text{e}}) ({\text{GGT}}) + 0.053 \times \mathrm{ WC } - 15.745}) }{(1+ {{\text{e}}}^{ 0.953\times {\text{log}}\left({\text{e}}\right)\left({\text{TG}}\right)+ 0.139\times \mathrm{ BMI}+ 0.718\times {\text{log}}\left({\text{e}}\right)\left({\text{GGT}}\right)+ 0.053 \times \mathrm{ WC } - 15.745 }) }\times 100$$

The FLI demonstrates an accuracy of 0.83 (95% CI: 0.82 to 0.84) in detecting fatty liver, as measured by the area under the receiver operator characteristic curve (AUROC). The FLI ranges from 0 to 100. A FLI score of < 60 indicates the absence of fatty liver, while a FLI score ≥ 60 indicates the presence of fatty liver, with good diagnostic accuracy^19^.

Participants' dietary information was obtained through face-to-face interviews using a 118-item semi-quantitative food frequency questionnaire (FFQ)^20^. The HBI score was calculated using Duffy and Dewey's approach^9^. The HBI score consists of ten categories, each with a different score range, including water (0–15 points), unsweetened tea and coffee (0–5 points), diet drinks including artificially sweetened beverages and non-caloric (0–5 points), natural fruit juice (0–5 points), alcohol (0–5 points), soft drinks and sweet coffee (0–15 points), total beverage energy (0–20 points), and meeting fluid requirements (0–20 points). The scores were based on the total amount of drinks consumed per day. The HBI score ranges from 0 to 100, with a higher cumulative score indicating better adherence to the healthier HBI pattern. In this study, the highest possible HBI score was 80 due to the unavailability of information on alcohol content and diet beverages^21^.

### Statistical analysis

All analyses in this study were carried out using Stata version 14.2 software (Stata Corp, College Station, TX, USA). The general characteristics, anthropometric indices, and biochemical factors of participants were presented as mean ± standard deviation and number (percentage), across tertiles of the HBI score. To compare differences across HBI tertiles, the one-way ANOVA test was used for continuous variables and the chi-square test was used for qualitative variables. Logistic regression analysis was conducted to explore the associations between FLI and HBI. Additionally, linear regression was utilized to determine the associations between AST, ALT, ALP, and GGT with HBI. The multiple models controlled for age, sex, SES, energy intake, physical activity, and smoking variables. For all analyses, a *P* value of < 0.05 with 95% confidence intervals (CIs) was considered significant.

### Ethics approval and consent to participate

The study was approved by the ethics committee of Kermanshah University of Medical Sciences (KUMS.REC.1394.318). All methods were carried out in accordance with relevant guidelines and regulations. All the participants were provided oral and written informed consent. This study was conducted by the Declaration of Helsinki.

## Results

The frequency of females in the third tertile, indicates a healthier beverage index was significantly higher than that of males. The average WC in the first tertile of HBI was higher than the third tertile (97.04 vs. 96.28 cm; *P* < 0.001). The mean VFA was also higher in the first tertile than in the third tertile of HBI (120.57 vs. 117.56 cm^2^; *P* = 0.047). The average FLI was significantly lower in the first tertile of HBI compared to the third tertile (47.83 vs. 45.77; *P* = 0.001). The average of FBS has decreased across tertiles of HBI (90.37 vs. 89.82; *P* = 0.014) (Table 1).

**Table 1:** Baseline characteristics of participants by healthy beverage index tertile.

Variables	Healthy beverage index			*P* value
	Tertile 1	Tertile 2	Tertile 3	
	Mean ± SD or n (%)			
Frequency	2130	2071	2172	
Age (years)	45.99 ± 7.89	45.91 ± 7.81	45.69 ± 7.63	0.276
Gender, n (%)				
Male	1013 (47.56)	857 (41.38)	997 (45.90)	< 0.001
Female	1117 (52.44)	1214 (58.62)	1175 (54.10)	
Residence, n (%)				
City	1176 (55.2)	1258 (60.7)	1307 (60.2)	< 0.001
Village	954 (44.8)	813 (39.3)	865 (39.8)	
Physical activity (Met h/day), n (%)				
Low (24–36.5)	580 (27.23)	613 (29.60)	651 (29.97)	0.003
Moderate (36.6–44.9)	1025 (48.12)	1044 (50.41)	1066 (49.08)	
High (≥ 45)	525 (24.65)	414 (19.99)	455 (20.95)	
Socioeconomic status, n (%)				
Q1 (lowest)	389 (18.26)	434 (20.98)	453 (20.86)	0.210
Q2	448 (21.03)	386 (18.66)	422 (19.43)	
Q3	436 (20.47)	420 (20.30)	423 (19.48)	
Q4	409 (19.20)	420 (20.30)	415 (19.11)	
Q5 (highest)	448 (21.03)	409 (19.77)	459 (21.13)	
Current smoker, n (%)	240 (11.39)	200 (9.80)	194 (9.03)	0.084
BMI, kg/m^2^	27.42 ± 4.58	26.80 ± 4.61	27.03 ± 4.52	< 0.001
WC, cm	97.04 ± 10.29	95.47 ± 10.43	96.28 ± 10.37	< 0.001
BFM, kg	33.68 ± 9.5	33.51 ± 9.5	33.35 ± 9.3	0.525
VFA, cm^2^	120.57 ± 51.3	116.98 ± 50.7	117.56 ± 49.9	0.047
AST, mg/dL	21.40 ± 8.11	20.80 ± 7.32	21.47 ± 9.60	0.017
ALT, mg/dL	24.72 ± 16.14	23.41 ± 12.56	24.37 ± 14.01	0.009
GGT, mg/dL	23.82 ± 22.50	22.15 ± 15.21	23.13 ± 17.54	0.016
ALP, mg/dL	193.37 ± 57.63	190.11 ± 52.90	190.65 ± 54.63	0.120
FLI	47.83 ± 26.95	44.13 ± 26.61	45.77 ± 26.50	0.001
LDL-C, mg/dL	112.20 ± 31.64	110.85 ± 31.11	110.82 ± 29.52	0.249
HDL-C, mg/dL	46.71 ± 11.29	47.36 ± 11.59	47.11 ± 11.40	0.176
TG, mg/dL	132.49 ± 77.81	125.98 ± 69.01	129.11 ± 72.48	0.016
TC, mg/dL	185.37 ± 37.71	183.41 ± 37.10	183.74 ± 35.50	0.181
FBS, mg/dL	90.37 ± 9.70	89.53 ± 9.28	89.82 ± 9.47	0.014
Energy intake (kcal/day)	2346.24 ± 726.3	2702.05 ± 716.0	2670.41 ± 705.1	0.001

*Based on one-way ANOVA and chi square tests.

BMI: Body mass index, VFA: Visceral fat area; BFM: Body fat mass ;WC: Waist circumference; HDL-C: High-density lipoprotein cholesterol, LDL-C: Low-density lipoprotein cholesterol, TG: Triglycerides, TC: Total cholesterol, FBS: Fasting blood sugar, AST: Aspartate transaminase, ALT: Alanine transaminase, GGT: Gamma-glutamyl transferase; ALP: Alkaline phosphatase.

The average sugar-sweetened beverages consumption in the high FLI group was higher than the low FLI group (737.90 vs. 767.89 mL/day; *P* = 0.023). Average low-fat milk consumption was higher in the low FLI group, although not statistically significant (*P* = 0.634). The average energy from beverages (%), met fluid requirement and fruit juice were also higher in the high FLI group than in the low FLI group, and this finding was statistically significant (*P* < 0.05). Additionally, the average HBI was significantly lower in the high FLI group (*P* = 0.014) **(**Table 2**).**

**Table 2:** Distribution of healthy beverage index component by group (NAFLD &Non-NAFLD).

Healthy beverage index component	Non-NAFLD	NAFLD	*P* value*
	(n = 4162)	(n = 2114)	
Water intake (mL/day)	2196.01 ± 1356.65	2378.70 ± 29.99	0.001
Low-fat milk (mL/day)	50.10 ± 67.71	49.20 ± 69.46	0.634
100% fruit juice (mL/day)	4.68 ± 13.43	6.08 ± 16.26	0.003
Sugar-sweetened beverages (mL/day)	737.90 ± 495.73	767.89 ± 496.28	0.023
% energy from beverages	9.15 ± 4.53	9.57 ± 4.59	< 0.001
Met fluid requirement (mL/day)	2532.98 ± 731.62	2593.96 ± 728.56	0.001
Healthy beverage index	56.10 ± 9.04	55.50 ± 9.03	0.014

*Based on t-test; NAFLD: non-alcoholic fatty liver disease.

The univariable analysis showed a significant association between high FLI and HBI. Individuals in the second and third tertiles of HBI had 18% and 12% lower odds of having high FLI compared to individuals in the first tertile, respectively. Furthermore, after adjustment for some variables, the odds of high FLI decreased by 28% (OR 0.72, 95% CI 0.63, 0.82) in the second tertile of HBI and by 21% in the third tertile (OR: 0.79, 95% CI 0.70, 0.91).

In the univariate linear regression model, it was found that in the second tertile of HBI, the mean ALT decreased significantly by 1.31 mg/dL, and in the third tertile, it decreased by 0.35 mg/dL, which was not statistically significant. A similar association was observed in the adjusted model for confounding variables.

Furthermore, in the univariate analysis, it was observed that in the second tertile of HBI, the average GGT significantly decreased by 1.65 mg/dL (β = − 1.65, 95% CI − 2.79, − 0.52), and in the third tertile, it decreased by 0.68 mg/dL (β = − 0.68, 95% CI − 1.80, 0.44), which was not significant. The same association was observed in the adjusted model (Table 3).

**Table 3:** Association between healthy beverage index and liver indices.

Variables	Healthy beverage index		
	Tertile 1	Tertile 2	Tertile 3
	OR (95% CI)		
Fatty liver index			
Unadjusted model*	1	0.82 (0.72, 0.92)	0.88 (0.78, 0.99)
Adjusted model*	1	0.72 (0.63, 0.82)	0.79 (0.70, 0.91)
Aspartate aminotransferase			
Unadjusted model**	0	-0.60 (-1.11, -0.10)	0.07 (-0.43, 0.57)
Adjusted model**	0	-0.40 (-0.91, 0.11)	0.07 (-0.43, 0.58)
Alanine aminotransferase			
Unadjusted model**	0	-1.31 (-2.18, -0.44)	-0.35 (-1.21, 0.51)
Adjusted model**	0	-1.12 (-1.96, -0.26)	-0.54 (-1.38, 0.28)
Gamma-glutamyl transpeptidase			
Unadjusted model**	0	-1.65 (-2.79, -0.52)	-0.68 (-1.80, 0.44)
Adjusted model**	0	-1.40 (-2.55, -0.24)	-0.73 (-1.86, 0.39)
Alkaline phosphatase			
Unadjusted model**	0	-3.26 (-6.61, 0.08)	-2.71 (-6.02, 0.58)
Adjusted model**	0	-3.78 (-7.20, -0.36)	-3.27 (-6.61, 0.07)

*By logistic regression analysis.

**By linear regression analysis.

Adjusted model: adjusted for age, sex, SES, energy intake, physical activity and smoking.

**Figure 2:**
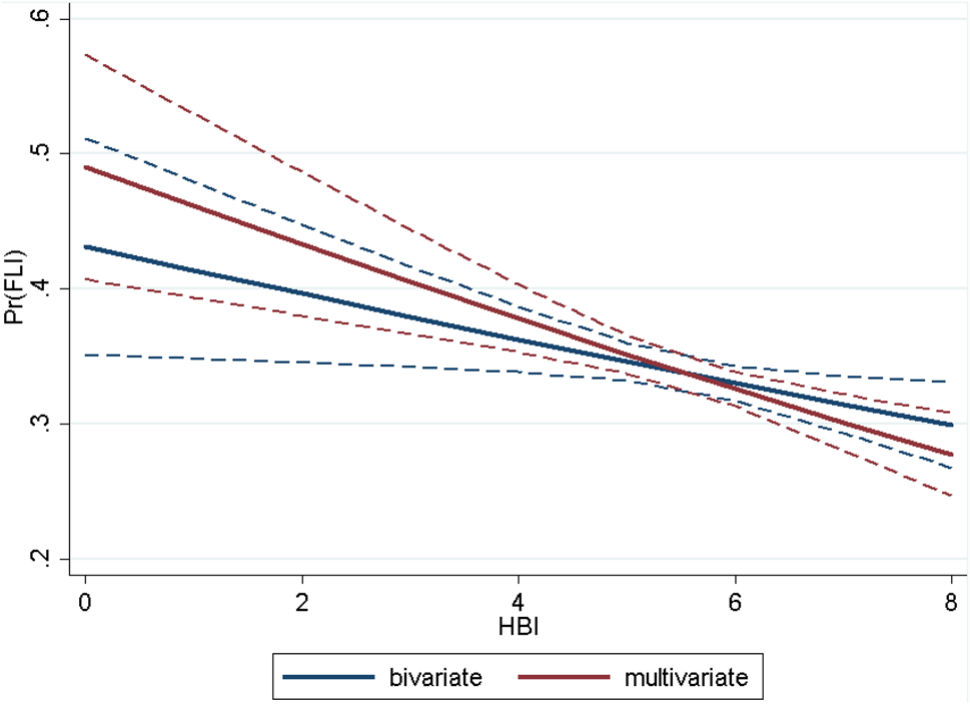
Association between healthy beverage index (HBI) and fatty liver index (FLI).

For more detailed information on the univariable and multiple associations between FLI and HBI (Fig. 2).

## Discussion

The study findings indicate an inverse association between high FLI and HBI. After adjusting for confounding variables, we observed a significant decrease in the odds of high FLI across HBI. Specifically, in the second tertile of HBI, the odds of high FLI was 28% less than in the first tertile, and in the third tertile, the odds were 21% less than in the first tertile.

Park et al.'s study reveals that frequent users of SSB have 2.53 times higher odds of NAFLD compared to non-users^14^. Similarly, Ma et al. found that regular consumption of SSB is associated with a higher risk of NAFLD, particularly in overweight and obese subjects. Furthermore, the consumption of SSB is positively correlated with ALT levels^22^. Two small case–control studies also indicate that people with NAFLD consume more SSB compared to controls without NAFLD, independent of overall obesity^23,24^. Based on the information provided, it appears that there is limited study specifically examining the relationship between the HBI and NAFLD. However, some studies have investigated the relationship between HBI and metabolic diseases such as type 2 diabetes, insulin resistance, and cardiometabolic diseases. These studies have found an inverse associated between the HBI and these conditions^7–9^. Therefore, the evidence suggests that healthy beverage may have a beneficial impact on the metabolic system and liver function.

In this study, we discovered that there was no relationship between GGT, ALT, ALP and AST levels with HBI. However, the Framingham cohort study observed a positive association between the SSB and ALT levels^22^. The average consumption of Natural fruit juice and SSBs in the group with NAFLD was significantly higher than in the other non-NAFLD. This finding confirms the intake of more fructose and sugar in the NAFLD group.

Several mechanisms have been proposed to explain how fructose may contribute to hepatic fat accumulation. The liver is the primary site for fructose metabolism, where it is converted to pyruvate/acetyl CoA and utilized as a substrate for the production of new fatty acids^25^. Unlike glycolysis, this process is not controlled by the primary rate-limiting enzyme, phosphofructokinase^26^. Additionally, fructose can activate sterol receptor element-binding protein 1c (SREBP-1c) and carbohydrate response element-binding protein (ChREBP)^27,28^, which are key transcription factors in lipogenesis. Another possible pathway is the inhibition of fructose fatty acid catabolism, which reduces β-oxidation activity in the liver^25,29^. Furthermore, there is a hypothesis that intermediate products, like diacylglycerols generated during the conversion of fructose to triglycerides, could contribute to insulin resistance and the subsequent accumulation of fat in the liver^30,31^. Scientific documents show that SSB consumption may increase the risk of hyperuricemia through a severe decrease in adenosine triphosphate (ATP)^32,33^, a process that may subsequently increase ALT levels. It is noteworthy that the association between SSB consumption and ALT levels may not be due to the accumulation of fat in the liver alone, as increased ALT may also be due to liver inflammation. Glucose is another major component of added sugars in SSB. Lanaspa et al. found that glucose may be converted to fructose via the polyol pathway in the liver, leading to fatty liver^34^. Because of the cross-sectional nature of the current study, it is not feasible to determine the potential role of the mentioned mechanisms.

In this study, the hypothesis regarding the association between beverages and NAFLD was supported. This study had several limitations. The main limitation of this study is that NAFLD was not diagnosed by ultrasound or magnetic resonance imaging (MRI) but was diagnosed using the FLI algorithm. Additionally, this study is cross-sectional, meaning that it cannot be a causal relationship. To further validate these findings, it is recommended to conduct longitudinal studies that can provide more robust evidence on the association between beverages and NAFLD. According to the method of completing the FFQ, we were unable to assess the consumption of tea and coffee due to the inability to differentiate between the sugar and sugar consumed with them. This study was conducted on a large group of Iranian adults and may not be generalizable to all age and geographical groups. It is important to replicate the study in other populations.

The large sample size was one of the strengths of the study. We tried to control for potential confounders, although we are unable to control for all variables in studies and have residual confounding estimates.

## Conclusion

The present population-based study provides evidence of the effect of beverages on liver function. The findings revealed a significant inverse association between HBI and NAFLD, indicating that individuals who consumed healthy beverages were less likely to have NAFLD. Therefore, it is recommended to consume healthy beverages and without added sugar. However, additional longitudinal studies are required to examine the association between beverage consumption and the development of NAFLD.

## Data Availability

The data analyzed in the study are available from the corresponding author upon reasonable request.
